# Extensive Self-Harm Scarring: Successful Treatment With Simultaneous Use of a Single Layer Skin Substitute and Split-Thickness Skin Graft

**Published:** 2012-05-21

**Authors:** Jodi Todd, Sara Ud-Din, Ardeshir Bayat

**Affiliations:** University Hospital of South Manchester NHS Foundation Trust, Faculty of Medical and Human Sciences, University of Manchester, Manchester Academic Health Science Centre; and Plastic and Reconstructive Surgery Research, School of Translational Medicine, Manchester Interdisciplinary Biocentre, University of Manchester, Manchester, UK

## Abstract

**Objective:** Deliberate self-harm resulting in extensive skin scarring is a difficult clinical problem and is commonly associated with physical and sexual abuse or a known history of mental illness. Immediate hospital attendance often addresses the acute wound and current psychological state of patients; however, ongoing regret of these resulting scars present a problem to the patient and clinician. Deliberate self-harm to the skin leaves permanent and socially unacceptable scars in anatomically conspicuous areas and recognizable to others. Therefore, the aim was to offer a treatment to change these scars to that of an unknown entity. **Methods:** Six patients with extensive linear scars covering most of the forearm received surgical reconstruction. Patients were female aged between 18 and 47 years. Each patient had a history of psychosocial problems, and each had undergone psychiatric treatment. After an in-depth consultation and a further clinical psychological assessment, each individual was deemed suitable for reconstructive surgery. Scars were excised from the forearm en block, removing the majority of the affected area. Simultaneous use of a single layer skin substitute was used, covered by an autologous split-thickness skin graft. Negative pressure wound therapy was then applied immediately for 2 weeks after surgery. **Results:** The original scars were successfully converted to a socially and cosmetically acceptable appearance. Postoperative infection due to negative pressure wound therapy failure in one patient was the only complication reported. **Conclusions:** This case series highlights the utility of an innovative treatment for patients with DSH scarring resulting in aesthetic, psychological, and functional benefits.

*Deliberate self-harm* (DSH) can be defined as intentional self-poisoning or self-injury.^1^^,^^2^ This can be inflicted through various methods, the most common being substance or drug ingestion and self-initiated cutting behavior. This not only presents ongoing problems and a constant reminder to the individual patient but also a major challenge to the clinician.

Rates of DSH have increased throughout the world in recent decades,^3^ and are most prevalent amongst those of adolescent age, more commonly females.^1^^,^^4^^,^^5^ Difficulties expressed by individuals at the time of DSH frequently include depression and anxiety, and are often related to alcohol and substance misuse as a result of suffering traumatic events such as physical or sexual abuse.^6^ Performing acts of DSH is thought to give individuals a sense of relief from a terrible state of mind.^3^

Scar treatment often involves a lengthy process and an initial period of observation is advised in order for the scar tissue to mature.^7^ Treatments can be invasive using interventions such as intralesional steroid injections or surgical revision; however, DSH scars are often flat and linear and therefore have no response to steroid injections, which are commonly reserved for the treatment of raised dermal scars.^8^ Surgical revision is difficult as the scars usually cover a large area consisting of many fine linear or stretched scars. Camouflage can be used as a temporary solution although patients soon feel that this is inadequate coverage. Topical silicone gels can also be used, primarily to address the symptoms of itch but will not completely eradicate these scars.^9^

There are currently limited treatment options available to modify the appearance of DSH scars. We present a case series of 6 patients with simultaneous use of a single layer skin substitute (Integra Life Sciences, Plainsboro, NY) and a split-thickness skin graft (STSG). The procedure was completed in a single stage, combined with immediate application of negative pressure wound therapy (NPWT). To our knowledge, there are no previous reports of this single stage treatment for use in DSH scar reconstruction.

## METHODS

A retrospective study on 6 patients was completed over a period of 12 months. All the patients were referred by their general practitioner to the specialist scar service in the department of plastic and reconstructive surgery at University Hospital of South Manchester, England, United Kingdom. Reasons for referral were usually due to the patient feeling unsatisfied about the appearance and nature of their visually conspicuous DSH scars. All 6 patients were female and white British with the exception of one, of black Afro-Caribbean origin. Similarly, the individuals all began DSH behavior at various times between the ages of 12 and 15 years, continuing for a minimum of 3 years to a maximum of 27 years. A range of sharp objects were used to inflict the DSH scars including items such as pencil sharpener blades and glass.

**Figure 1:**
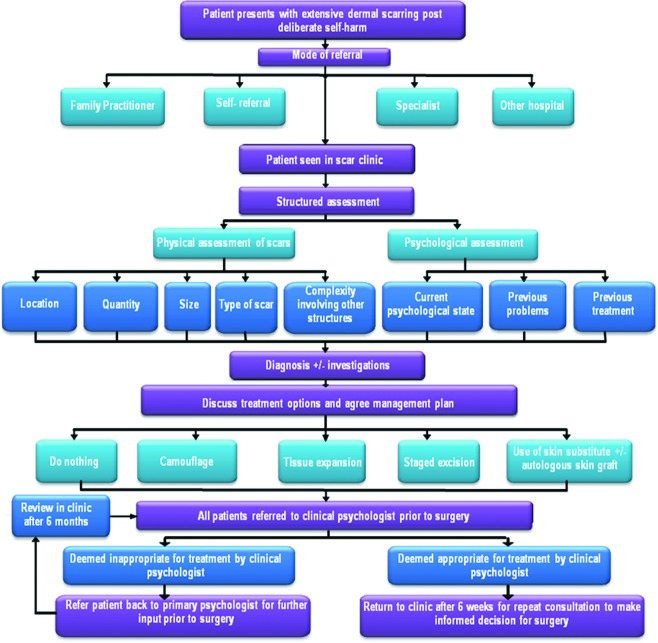
Deliberate self-harm patient assessment flowchart. Flowchart depicting the process for all patients to be considered for deliberate self-harm scar reconstructive surgery.

Each individual had an initial in-depth consultation covering the history of their scars, psychological state and reasons for causing the scars. All treatment options including the alternatives as seen in the treatment pathway (Fig 1) were explained in detail. It was of paramount importance to explain and describe the final outcome and appearance of their new skin following treatment. It was emphasized that their scars would not disappear completely but would be replaced by a different type of scar, not exactly resembling the patient's own native skin. After the consultation, the patient was given a minimum period of 6 weeks to consider the options. Implications explained to the patient included risks of anesthesia, risk of infection, graft loss, altered sensation to the skin, and altered pigmentation as well as redness at the edge of the graft with a risk of raised dermal scarring. In addition, the need for frequent dressing changes, regular outpatient visits, relatively extended healing period due to the delicate nature of the skin grafting, wearing a pressure garment following surgery and further risk of hyperpigmentation if exposed to sunlight were explained to and discussed with the patient. All patients were routinely required to go to an independent psychologist based at the clinical psychology unit at University Hospital of South Manchester for a psychological assessment prior to any surgical intervention. Any details of current psychological state were requested from the patient's own psychiatrist/psychologist.

Some patients presented with scars to bilateral arms; however, due to the grafting and restrictions using the arm postoperatively, treating one arm only was considered feasible at the first surgical intervention.

**Figure 2:**
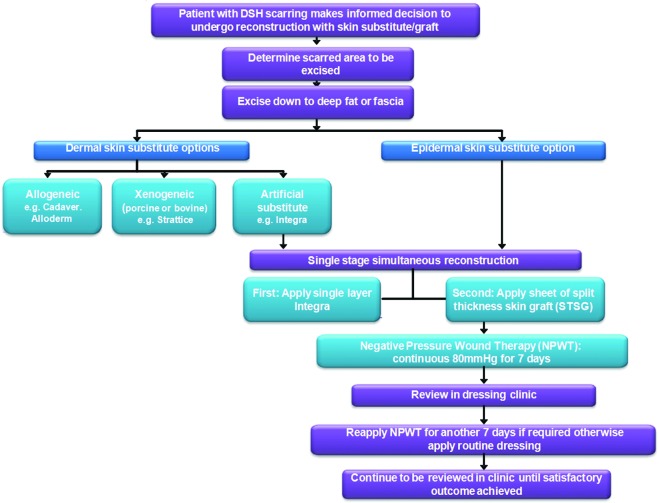
Surgical pathway flowchart. Flowchart demonstrating the surgical pathway for patients deemed suitable for reconstructive surgery on their deliberate self-harm scars.

**Figure 3:**
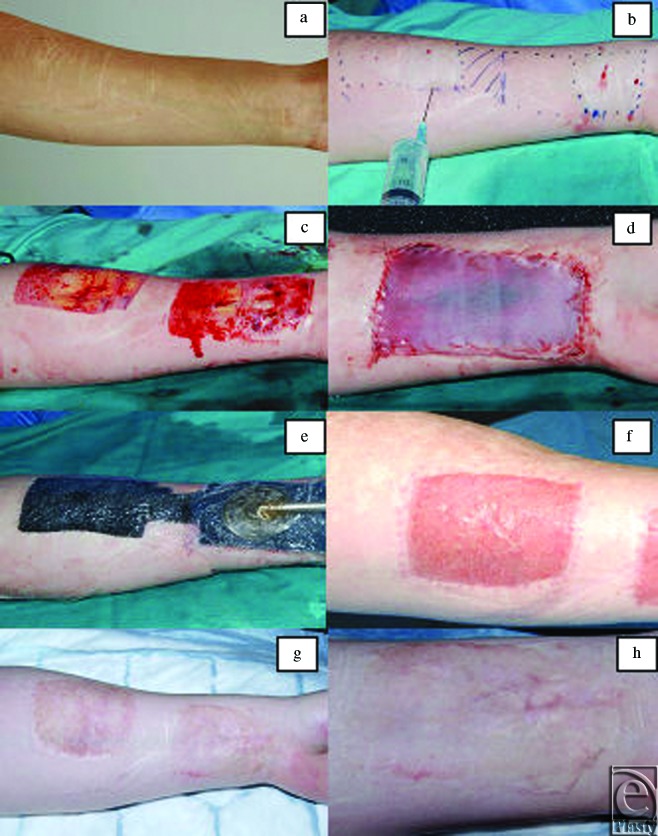
Images of case 1: surgical procedure and outcome. (*a*) Deliberate self-harm scars before any surgical intervention. (*b*) Area selected for surgical excision and local anesthetic administered. (*c*) Scars surgically excised in a block down to deep bleeding fat. (*d*) Integra and split-thickness skin graft secured into place with sutures. (*e*) Negative pressure wound therapy immediately applied. (*f*) 3 months postreconstructive surgery. (*g*) 15 months postreconstructive surgery. (*h*) 15 months postreconstructive surgery (close up).

**Figure 4:**
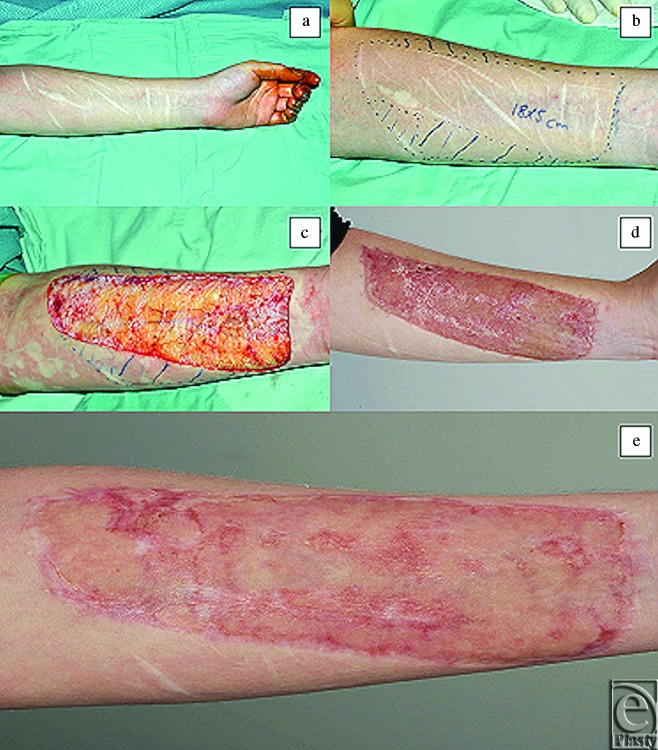
Images of case 5: surgical procedure and outcome. (*a*) Deliberate self-harm scars prior to any surgical intervention. (*b*) Area selected for surgical excision. (*c*) Scars surgically removed in a block down to deep bleeding fat. (*d*) 3 months postreconstructive surgery. (*e*) 8 months postreconstructive surgery.

**Figure 5:**
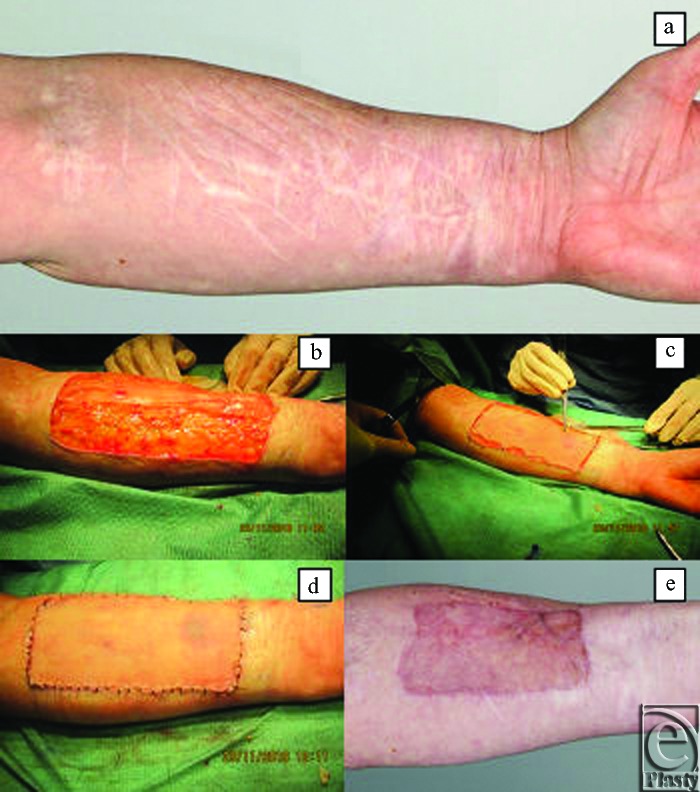
Images of case 6: surgical procedure and outcome. (*a*) Deliberate self-harm scars prior to any surgical intervention. (*b*) Application of Integra over deep bleeding fat. (*c*) Anchoring sutures to the Integra and split-thickness skin graft applied simultaneously. (*d*) Integra and split-thickness skin graft securely sutured into place. (*e*) 4 months postreconstructive surgery.

Every patient consented and underwent surgery under a general anesthetic as a day case (process depicted in a flowchart in Fig 2). The surgical method is also shown in the images of some of the cases, which are shown in Figures 3–5. The area of DSH skin scarring was marked and excised as a block of dermis from the affected forearm down to deep bleeding fat (Figs 3–5), all scar tissue was removed in total. Meticulous hemostasis was achieved prior to the application of single layer dermal skin substitute (Integra Life Sciences, Plainsboro, NJ), measured to the size of the defect and secured with several anchoring 5/0 vicryl rapide (Ethicon, Johnson & Johnson, Somerville, NJ), suture material. This was immediately followed by application of a sheet (non-meshed or fenestrated) of STSG harvested from either the thigh or the upper inner aspect of the affected arm. The choice of donor site was discussed at length prior to the procedure. Ipsilateral upper inner arm gives the advantage of confining the wounds to one limb; however, the skin can be thinner and may take longer to heal following graft take. On the contrary, the donor site from the lower leg often has thicker skin and potentially offers a quicker healing period but would leave 2 wounds on 2 limbs with a possibility of donor site wound complications. This sheet of STSG was sutured into place with 6/0 prolene (Ethicon, Johnson & Johnson, Somerville, NJ) and 5/0 viryl rapide (Ethicon, Johnson & Johnson, Somerville, NJ) suture material and skin adhesive (Dermabond, Johnson & Johnson, New Brunswick, NJ). Negative Pressure wound therapy foam dressing (VAC therapy, KCI, San Antonio, TX or Renasys, Smith & Nephew St Petersburg, FL) was then applied and secured at -80 mm Hg continuously as shown in Figure 3. This remained in place for 7 days prior to a wound check and new NPWT foam dressing being re-applied. Further dressing changes occurred every 7 days, unless required sooner, with the NPWT remaining in-situ for a total of 2 weeks. After this period, a soft silver impregnated foam dressing (Polymem silver, Ferris Mfg Corp, Burr Ridge, IL) was applied. Following complete healing of the wound, a silicone-lined pressure garment (Silimed, Botafogo, Rio de Janerio, Brazil) was applied for long-term protection for a minimum of 3 months. Donor site dressing changes took place every 3 to 5 days postoperatively unless required sooner. Again, a (Polymem silver, Ferris Mfg. Corp, Burr Ridge, IL) dressing was applied to the donor site wound until fully healed.

### Case 1

A 32-year-old woman with a history of psychological problems spent 1 year in an adolescent psychiatric unit and received psychotherapy for an eating disorder. Acts of DSH were carried for 7 years between the ages of 14 and 21 years, including cutting with objects such as glass, and blades and self-poisoning. All cuts were made to her left nondominant upper and lower arm alone, leaving her with numerous scars. Scar reconstruction was carried out on her left forearm.

### Case 2

A 20-year-old woman presented with a history of moderate depressive disorder and personality difficulties. She was taking antidepressants and had a history of DSH for 5 years, between the ages of 12 and 17 years. She had multiple lacerations that were made with razor blades to bilateral forearms. Scar reconstruction was performed on her left forearm.

### Case 3

A 32-year-old Afro-Caribbean female presented, on antidepressants for depression. With a 9-year history of DSH between the ages of 14 and 23 years, razor blades were used to both arms and she presented with linear scars to both forearms, one of which had become very stretched and unsightly. Scar reconstruction was performed on her right forearm.

### Case 4

At the age of 18 years, the youngest patient had a 3-year history of DSH from the age of 13 to 16 years. Scars were made with razor blades and noted to bilateral forearms and upper arms. Scar reconstruction was to the left forearm, excising the block of scarred tissue and replacing this with single layer Integra and an STSG taken from the left upper inner arm. She developed a pseudomonas aeruginosa infection postoperatively due to failure of the NPWT dressing. An explanation for this may be due to the extensive nature of her scars covering her entire volar forearm, which required the NPWT to be applied across both her wrist and elbow crease. A lack of sufficient seal led to multiple attempts to fix this and a final abandonment of using NPWT. This infection was treated with oral antibiotics and frequent dressing changes and was resolved within 7 days. This resulted in a 50% graft loss, which was subsequently followed with reapplication of STSG to areas of lost graft take. No further NPWT was used in the postmanagement of this case.

### Case 5

A 28-year-old female presented who suffered from bipolar disorder and depression. She had a history of being in foster care as an adolescent with a number of psychosocial issues. She had a 9-year history of DSH between the ages of 13 and 22 years including overdose as well as self-cutting with razor blades and any other sharp objects found. Multiple linear scars were seen to bilateral arms with the left arm being more severe. Scar reconstruction was performed on her left forearm.

### Case 6

At 47 years of age, slightly older than the average patient seen in clinic for DSH, she suffered from bipolar disorder and depression, was under a psychiatrist, and was taking antidepressants. She had a long history of DSH stemming back for 27 years between the ages of 15 and 42 years. This included self-poisoning, jumping from a building as well as cutting of the arms and abdomen. She used razor blades, pencil sharpeners, cigarettes, and knives as some of the objects to cause cutaneous lacerations. Extensive scars to both forearms were noticeable in vertical and horizontal directions covering the whole circumference of both arms. Scar reconstruction was given to the area with most scar tissue on the left arm.

## RESULTS

All 6 patients received the same treatment in terms of surgical technique and postoperative management, of which there was only one reported problem. One patient developed an infection postoperatively due to failure of the NPWT dressing. Full wound closure was witnessed in all patients, although at different time periods ranging between 2 to 4 weeks. Recognizable evidence of DSH scars were removed and replaced with new skin that appeared more similar to a well-healed superficial dermal burn scar or one caused by a well-healed traumatic dermal abrasion as demonstrated in Figures 3–5. In addition, none of the resultant new skin bore any resemblance to the original DSH scars. There was a variation in the level of pigmentation (various degrees of hyperpigmentation) observed in the reconstructed area in comparison with the patient's native normal skin.

In the case of extensive dermal scarring affecting both volar and dorsal aspects of the forearm, the senior author chose to treat these in stages by removing either volar or the dorsum in the first instance before addressing the other side. A similar staged surgical reconstruction approach was taken for patients suffering from scars on bilateral forearms and/or scars in the forearm versus the upper arm. Following in depth discussion with the patient, the area of scarred skin most obvious to the patient was removed in a single block in the first instance. All patients reported that they were satisfied with the scars and those with scarring to bilateral arms were keen to proceed with treatment to these areas in the same way.

## DISCUSSION

This case series highlights the utility of an innovative surgical treatment approach of using single stage dermal reconstruction for managing patients with clinically challenging DSH scarring. This has resulted in esthetic, psychological, and functional benefits to patients, who had previously been overlooked and suboptimally managed.

Previously, a case series of 2 patients was reported in 2008,^10^ which described using an artificial skin substitute, two-layer Integra (Integra Life Sciences, Plainsboro, NJ) and vacuum-assisted wound closure in the first stage, followed by application of an STSG 3 weeks later in the second stage. This 2-stage reconstruction required patients to have surgery on 2 separate occasions and extended the recovery and healing period as well as the additional cost of having a second operative procedure. By the use of this single stage reconstruction and application of a portable NPWT device, we allowed patients to be mobile on the same day as surgery. This approach reduced the cost of inpatient stay and the additional cost of a separate surgical procedure.

Although a treatment to these individuals has been offered, the initial problem of DSH should not be overlooked. DSH behavior is prevalent in the adolescent years and rates have increased throughout the world in recent decades.^3^ DSH is considered to be more common in females,^11^^,^^12^ although interestingly in a study carried out by Rashid and Brennen,^13^ which looked at patient admittance to hospitals following DSH, the number of males to female ratio was 2:1. Reasons for this were not defined; however, an assumption can be made that males inflict deeper cuts than females. Only 41 patients were included, and it did not reflect national trends amongst those who were not admitted into hospital. Anecdotal evidence suggests that DSH cutting rarely causes deep structural injury to upper limbs. In a study completed by Hawton et al,^14^ it was found that of 6020 pupils who reported DSH only 12.6% reported this to hospital or sought medical attention. Similar large community studies have shown that the majority of self-harmers do not receive any medical attention for their behavior.^5^^,^^15^^,^^16^ Pages et al^17^ reported how most hospital-admitted self-harmers attend due to self-poisoning rather than self-cutting. Therefore, there may be an underestimate of individuals with minor cuts due to DSH presenting to clinical practice.

Motives for DSH are varied amongst different groups of individuals; however, the most common factor reported is depression followed by anxiety.^18^^-^20 Our study of these cases is in agreement with Scoliers' study in 2009, which confirmed that each individual with a history of DSH has experienced a period of traumatic events. Other causal factors include low self-esteem^14^; being victim of bullying, alcohol, and substance misuse; and a strong association following physical and sexual abuse.^4^ In a case control study completed by Mahadevan et al,^1^ it was shown that the majority, 52.2% of pupils from the 261 university students, questioned whether academic study was the sole influencing factor in committing DSH.

It has been suggested that individuals select the forearm for manipulative reasons in which they have attention-seeking dimensions, hoping to stimulate care and communication from others.^3^ In addition, to the ease of anatomical access, self-reports from patients have indicated that relief is felt from visually observing the action of self-harm.

There are currently no treatments available for eradiation of cutaneous fine line flat scars. Simple surgical revision such as an elliptical excision will result in a similar scar, therefore, deemed unsuitable. Multiple or serial staged excisions of these scars are not ideal, because often these scars cover a large diameter and surface area of the forearm. Therefore, multiple excisions may result in an hour glass deformity with significant residual scars, which would still require removal. In addition, tissue expansion is not ideal in the forearm, due to limited volume of tissue that can be expanded. Camouflage is often advised although takes time to apply and is easily obliterated when engaging in routine daily activities. Spending time and effort trying to disguise these scars is frustrating to the individuals, and while treatments may reduce scars in size and improve their appearance they never remove them completely.

Living with scars can be challenging in a social environment that values physical attractiveness^21^^,^^22^ and up to half patients living with a disfigurement suffer concerning levels of anxiety, social avoidance and reduced quality of life.^23^ Brown et al^24^ demonstrated through semistructured interviews that the majority of patients (56%) were dissatisfied with the appearance of their scars. They felt abnormal and thought they were unsightly acting as a constant reminder of the causative event. This study was completed on patients who had suffered scars from various ways and was not subjective to DSH scars. To our knowledge, there has not been a quality of life (QoL) study completed on DSH scarring.

DSH scars are difficult to explain to others as an accident and from feedback from our patients if they were able to, they would feel more confident about integrating into society due to feelings of embarrassment and shame. These scars, especially on the arms, are very recognizable to others and are difficult to cover in social situations.

A more objective study would be justified for the future. A study to look closely at the QoL for these patients pre- and posttreatment would be helpful to determine the extent this has improved patients lives. This treatment option has allowed patients to change their DSH scars to those of an unknown entity. For these patients, it is not the cosmetic outcome that is most important or the removal of the scar itself but the disguise of the DSH behavior they once experienced.

In conclusion, this particular surgical treatment option (previously unreported) has offered an innovative approach to patients with DSH scarring to alleviate them from the constant reminder, not only of the physical scars but the psychological scars they represent. This effective therapeutic approach has provided an enhanced QoL to these patients as evidenced from our cohort of cases.
